# Navigating liver toxicity in the age of novel oncological agents

**DOI:** 10.1016/j.jhepr.2025.101473

**Published:** 2025-06-21

**Authors:** Mar Riveiro-Barciela, Eleonora De Martin

**Affiliations:** 1 Liver Unit, Internal Medicine Department, Hospital Universitari Vall d’Hebron, Vall d’Hebron Barcelona Hospital Campus, Barcelona, Spain; 2 Universitat Autònoma de Barcelona, Spain; 3 Centro de Investigación Biomédica en Red de Enfermedades Hepáticas y Digestivas (CIBERehd), Instituto de Salud Carlos III, Madrid, Spain; 4 European Reference Network on Hepatological Diseases (ERN RARE-LIVER); 5 APHP, Hôpital Paul Brousse, Centre Hépato-Biliaire Henri Bismuth, INSERM Unit 1193, Universite Paris-Saclay, Villejuif, France

**Keywords:** drug-induced liver injury (DILI), hepatotoxicity, checkpoint inhibitors, immunotherapy, checkpoint inhibitor-induced liver injury (ChILI), immune-mediated hepatitis, protein kinases inhibitors (PKIs), trastuzumab, CDK 4/6 inhibitors, antibody-drug conjugates (ADCs)

## Abstract

The advent of novel oncological therapies, including immune checkpoint inhibitors, antibody-drug conjugates, and protein kinase inhibitors, has revolutionised cancer treatment by significantly improving patient survival across a range of malignancies. However, these advances have been accompanied by the emergence of new and often unpredictable adverse events, among which hepatotoxicity represents a growing clinical challenge. In this review, we provide a comprehensive synthesis of current knowledge on liver injury associated with these three key classes of oncological agents, with a particular focus on mechanisms of action and hepatotoxicity, clinical presentation, and management strategies. Given the expanding use of these agents, both as monotherapies or in combination regimens, this topic is of pressing relevance to hepatologists and oncologists alike. As combination therapies become increasingly common, the complexity of drug–liver interactions and their implications for patient safety demand greater interdisciplinary awareness and collaboration. This review advocates for a pragmatic approach to the management of drug-induced liver injury in patients with cancer, underscoring the critical need to balance hepatic preservation with the imperative of maintaining oncological efficacy in this uniquely vulnerable population. By addressing an emerging area of clinical importance, we aim to stimulate further research on oncological hepatotoxicity, a phenomenon that is poised to become increasingly prevalent in routine clinical practice.

## Graphical abstract

**Figure:**
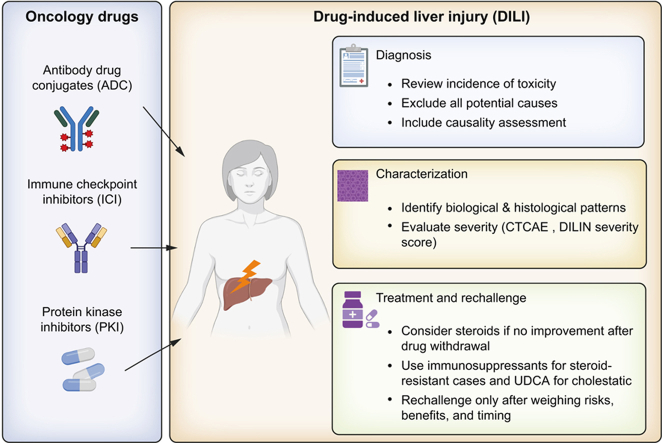

**Key points**

• The advent of new treatment options, such as immune checkpoint inhibitors, protein kinase inhibitors and antibody-drug conjugates, has significantly modified the prognosis of advanced and metastatic tumours.
• Drug-induced liver injury (DILI) is a diagnosis of exclusion, making a systematic evaluation of alternative causes essential – especially in severe and complex clinical cases.
• Though the CTCAE is the most widely used severity scale in oncology, bilirubin and INR (international normalised ratio) should always be addressed when DILI is suspected.
• The majority of severe DILI cases can be managed by temporary interruption of the causative drug if bilirubin and INR are normal.
• Corticosteroids are the main pillar of therapy in DILI cases presenting with increased bilirubin levels.
• Although rare, acute liver failure can develop as a result of these new drugs. Rechallenge is not recommended after a severe DILI episode, though this decision should be individualised, especially in patients with metastatic disease.

## Introduction

Over recent decades, the field of oncology has undergone a revolution with the discovery and introduction of new therapies that have dramatically improved the prognosis of some of the cancers with the highest mortality rates. The development of immune checkpoint inhibitors (ICIs) and their profound impact on survival have established immunotherapy as the first-line treatment for most advanced-stage cancers and as adjuvant therapy for several of the most prevalent malignancies, including breast cancer and non-small cell lung cancer.^1^ However, ICIs are not the only novel therapeutic modality. In recent years, important improvements have been made in antibody-drug conjugates (ADCs), which deliver cytotoxic agents directly to tumour cells by linking them to antibodies targeting tumour-specific antigens.^2^ Moreover, new small molecule protein kinase inhibitors (PKIs) have appeared, *e.g.* the cyclin-dependent kinase 4/6 inhibitors (CDKi), which are the first-line therapy for metastatic human epidermal growth factor receptor 2 (HER2)-negative breast cancer and have recently been approved by the EMA as adjuvant therapy for early breast cancer at high risk of recurrence.^3^

Although these drug classes target specific tumour antigens or signalling pathways, they can still cause side effects. In parallel with the exponential use of these new treatments, associated-adverse events, including liver toxicity, are increasing. In this context, recent real-world data from a tertiary hospital, including 106 DILI cases, revealed that antineoplastic agents were the most common cause, accounting for 26% of referrals.^4^ Thus, hepatologists need to be aware of the importance and general management of this toxicity. In this review, we aim to summarise current knowledge on the pathogenesis of hepatotoxicity induced by new oncological treatments and describe their current management, including the possibility of rechallenge after a first episode of DILI.

## General diagnostic and treatment considerations in oncological patients with DILI

When a drug is suspected to be the underlying trigger of liver test abnormalities, the first step in causality evaluation is to exclude alternative causes of hepatic injury,^5^ including concomitant medications and herbal supplements.^6^^,^^7^ Specific tools such as the RUCAM (Roussel Uclaf Causality Assessment Method) or more recently the RECAM (Revised Electronic Causality Assessment Method) are helpful in assessing causality and ease differential diagnosis, particularly in patients receiving multiple drugs.^8^ A thorough assessment of medication history is essential, particularly regarding the timeline of drug initiation and discontinuation, as a previously administered drug may remain active and continue to exert hepatotoxic effects. Moreover, it is crucial to exclude progression of the underlying tumour as the cause for aminotransferase elevation. For instance, in patients receiving ICIs, liver metastases are more commonly the cause of elevated alanine aminotransferase (ALT) levels than DILI.^9^ Ruling out viral hepatitis is also important, as it is frequently identified as an alternative diagnosis in cases of acute hepatitis.^10^ Along these lines, it should be stressed that, as for those undergoing chemotherapy, hepatitis B screening is recommended before initiating these new therapies. Although data regarding the risk of hepatitis B reactivation are still scarce for some of these drug families, in the case of immunotherapy, antiviral prophylaxis is needed for HBsAg-positive patients.^11^ For HBsAg-negative/anti-HBc-positive individuals, the risk of hepatitis B reactivation with ICI or PKI treatment is considered low^12^ and, therefore, only periodical HBsAg and HBV-DNA monitoring is recommended without antiviral prophylaxis, except in those with detectable HBV-DNA.^11^

Following a meticulous diagnostic work-up, a liver biopsy may be warranted in cases where the diagnosis remains unclear or when liver function fails to improve despite discontinuation of the suspected agent.^13^ A biopsy is particularly useful for elucidating the pattern of liver damage, especially when multiple aetiologies are considered.^14^ Moreover, although there is no specific histological pattern, some findings have been commonly reported in patients receiving anti-programmed cell death-1 (anti-PD1; lobular hepatitis) or anti-cytotoxic T-lymphocyte antigen 4 (anti-CTLA-4; granulomatous hepatitis, endotheliitis) agents, or some ADCs, such as gentuzumab (sinusoidal obstruction syndrome).^15^^,^^16^ In severe cases or instances of corticosteroid non-responsiveness, such as ICI-induced hepatotoxicity, a biopsy is recommended as part of the causality assessment.^17^^,^^18^

**Fig. 1:**
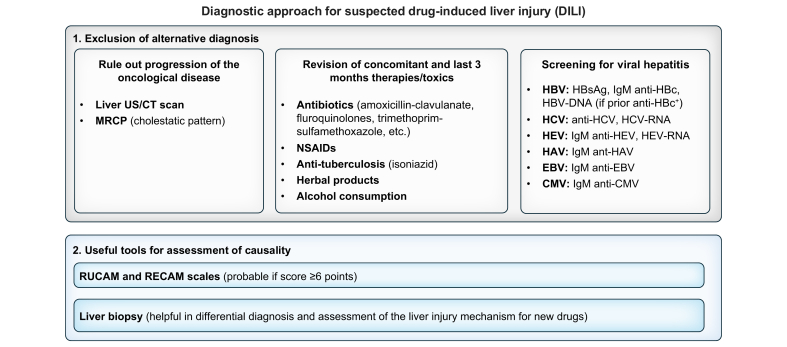
Diagnostic approach for suspected DILI. Since DILI is a diagnosis of exclusion, the first step is to rule out alternative causes of ALT increase, mainly progression of the tumour, as well as to screen for viral hepatitis and review concomitant drugs. In this regard, the RUCAM and RECAM scales could be useful tools for assessment of causality in case of multiple causative drugs. Liver biopsy could be helpful, especially if progression of the disease has not been completely discarded or in patients receiving new drugs whose liver injury mechanism remains unknown. ALT, alanine aminotransferase; DILI, drug-induced liver injury; MRCP, magnetic resonance cholangiopancreatography; NSAIDs, non-steroidal anti-inflammatory drugs; RECAM, Revised Electronic Causality Assessment Method; RUCAM, Roussel Uclaf Causality Assessment Method.

Liver function tests should be monitored closely following dose reduction or discontinuation of the suspected drug. In moderate cases, evaluations should be conducted every 48–72 h, whereas in severe cases – characterised by a decline in prothrombin time or an increase in bilirubin levels – daily monitoring is advised. If liver function does not improve despite drug withdrawal or dose adjustment, alternative therapeutic interventions must be considered, as summarised in Fig. 1.

At present, no specific treatments exist for anticancer drug-induced hepatotoxicity. Nevertheless, corticosteroids are frequently used in clinical practice and have shown beneficial effects in cases of moderate/severe DILI, excluding those with acute liver failure (ALF).^19^^,^^20^ They are the primary treatment for checkpoint inhibitor-induced liver toxicity (ChILI) and have also demonstrated efficacy in cases of CDKi-induced hepatotoxicity,^21^ where an immune-mediated mechanism is suspected. However, the optimal timing, dosage, and duration of corticosteroid therapy remain subjects of ongoing debate. Additionally, second-line immunosuppressive agents such as mycophenolate mofetil (MMF) or tacrolimus have been successfully utilised in refractory ChILI cases.^22^ Ursodeoxycholic acid has been employed with some success in cases of cholestatic DILI.^23^

In the rare instance of ALF, liver transplantation (LT) is generally contraindicated due to the presence of active malignancy. Although of limited efficacy, non-specific therapies may be considered in selected cases. Among these, extracorporeal liver support systems, including devices such as the MARS (molecular adsorbent recirculating system), remain the most widely utilised, although they are not recommended in patients who are not candidates for LT.^24^ Additionally, some hepatoprotective drugs such as intravenous N-acetylcysteine have demonstrated potential benefit in the early stages of liver failure, though more research is needed in the oncological setting.^25^^,^^26^ Finally, therapeutic plasma exchange has shown a positive effect on transplantation-free survival.^27^^,^^28^

Unlike other forms of DILI, in the context of cancer therapy, drug rechallenge is often necessary. This decision requires a careful balance between the risk of hepatitis recurrence and the overall benefit of continued oncological treatment.^29^ Certain anticancer agents carry a low-to-moderate risk of recurrence upon reintroduction, with some reports of successful rechallenge without further hepatic injury.^30^ When rechallenging in the setting of combination therapy, it is advisable to reintroduce agents sequentially to better identify the causative agent and minimise hepatotoxic risk. Where feasible, a switch to a different drug class is preferable, although in some instances, the absence of cross-toxicity allows for the continued use of another agent within the same class.^21^

## Checkpoint inhibitor-induced liver injury

### Mechanism of action and approved indications

The discovery of immune checkpoint inhibitors was a watershed in modern medicine, as recognised by the awarding of the Nobel Prize in 2018 to Professor James Allison and Professor Tasuku Honjo.

Briefly, the underlying mechanism of ICIs involves blocking the co-inhibitory signalling pathways that are overexpressed by tumoural cells to evade *cancer immunosurveillance*. This blockade restores the lytic effect of CD8+ cytotoxic T lymphocytes against the tumoural antigens expressed in cancerous cells.^31^^,^^32^ The most commonly targeted checkpoints are CTLA-4 and PD1 or its ligand PD-L1,^33^ though novel checkpoints have been described recently, such as TIGIT (T cell immunoglobulin and ITIM doming), LAG3 (lymphocyte activation gene 3 protein) and TIM-3 (T cell immunoglobulin and mucin domain 3).^34^

However, the negative regulatory pathway exploited by tumour cells is the same one that protects cells presenting autoantigens from immune-mediated destruction. This upregulation of cytotoxic T-lymphocyte activity as a result of ICI exposure may result in enhanced responses against autoantigens, resulting in immune toxicities known as “immune-related adverse events” (irAEs).^17^^,^^18^

The significant impact of ICIs on overall survival rates has led to immunotherapy becoming a first- or subsequent-line treatment for the majority of advanced and metastatic solid cancers.^1^ Furthermore, positive results with ICIs as adjuvant therapy – to reduce the risk of relapse in resectable tumours – have led to EMA approval of their use for non-small cell lung cancer, triple-negative breast cancer, bladder cancer and melanoma.^1^ EMA-approved ICIs are summarised in Table S1.

For certain tumour types, ICI monotherapy is ineffective and the combination of two or more ICIs, or ICIs combined with other oncological therapies – such as chemotherapy, targeted therapy, radiation, intratumoural therapies or other immunomodulators – are needed. These strategies may enhance oncological response, but they are usually associated with an increased risk of developing DILI.^35^

### Incidence and DILI pattern

ChILI has been described in 0.67-37% of patients treated with ICIs.^17^ The reported incidence of hepatitis depends on its severity and the number and type of ICIs used, ranging from 19-37% for grade 1 hepatitis in those treated with the combination of anti-PD1 and anti-CTLA-4 agents, to 0.67-3.0% for severe hepatitis in patients receiving monotherapy with anti-PD1 or anti-PD-L1 agents.^36^ Among patients treated with ICI monotherapy, the risk of severe ChILI is higher in those receiving an anti-CTLA-4 (4-9%) agent compared to an anti-PD1/PD-L1 agent (1-4%).^36^ Most cases are completely asymptomatic or are accompanied by non-specific symptoms such as asthenia, abdominal pain or fever.^37^ Therefore, patients are usually diagnosed following the check-up performed prior to the next cycle of immunotherapy. Only in very isolated cases does jaundice appear as the first sign of liver toxicity.^38^

ChILI usually arises during the first 4-12 weeks of immunotherapy, although cases have been described after the first cycle, as well as during the first 6 months after its discontinuation.[37], [38], [39] Several studies have reported that the hepatocellular pattern is the most common biochemical pattern of ChILI (50-60%), followed by mixed (20-30%) and cholestatic (20%).^37^^,^^40^^,^^41^

In both American and European Oncology guidelines, the severity of ChILI is defined according to the CTCAE and based on aminotransferase levels, with cases presenting with aspartate aminotransferase (AST) and/or ALT of ≥5x the upper limit of normal (ULN) or baseline being classified as severe.^17^^,^^18^ Nonetheless, since this scale omits the use of bilirubin and clotting, some guidelines recommend the use of the *International DILI Expert Working Group* (IEWG)^42^ or the *US DILI Network*^43^ scores for proper assessment of prognosis in patients with ChILI.^44^ A recent publication including 100 patients with ChILI reported a lower rate of severe cases when these scores were used instead of the CTCAE, a classification that presented a poorer correlation with the later development of clinical outcomes.^45^ Table S2 summarises the different scores for severity assessment in patients with DILI or ChILI.

Regarding risk factors, to date, it is impossible to predict which patients will develop irAEs, including ChILI, when receiving treatment with immunotherapy. However, it is known that some factors are associated with a higher risk of developing liver injury, such as anti-CTLA-4 agents, especially when combined with an anti-PD1 or anti-PD-L1 agent.^46^ Previous treatment with immunotherapy has also been associated with a higher risk of ChILI in case of retreatment.^47^ Moreover, the use of concomitant chemotherapy or kinase inhibitors has been linked with higher rates of toxicity, including ChILI (27%–29% for any grade, 13%–15% for grade >3).^36^

Other factors that have also been related to a higher risk of ChILI are female sex^40^^,^^48^ and the presence of elevated baseline levels of aminotransferases.^48^ Recently, some genetic variants associated with the immune response (*EDIL3, SAMA5A, GABRP, SMAD3, CD274* and *SLCO1B1*) have been linked to a higher risk of developing ChILI.^40^^,^^49^ A history of immune-mediated liver disease – such as autoimmune hepatitis or chronic cholestatic disease (primary biliary cholangitis or primary sclerosing cholangitis) – was not associated with an increased risk of irAEs in an international multicentre retrospective study.^50^ Neither the presence of liver metastases or baseline autoantibodies, such as antinuclear or anti-smooth muscle antibodies, have been associated with a higher incidence of ChILI in real-life cohorts.^51^

A unique scenario is hepatocellular carcinoma (HCC), a tumour that in more than 90% of cases develops in patients with underlying cirrhosis. Even though the overall risk of immunotherapy-related adverse events seems similar to that in other solid-organ tumours,^52^ the rate of ChILI is higher. For instance, in a cohort of patients with HCC receiving combination therapy with atezolizumab (anti-PD-L1) and bevacizumab (n = 375), the incidence of any-grade ChILI was 22.1 per 100 patient-years compared to a rate of 2.1 per 100 patient-years in those on monotherapy with an anti-PD1 agent for other solid-organ tumours (n = 459) (*p* <0.001).^53^ Fortunately, this increased risk of ChILI is linked to low rates of liver decompensation (7.0%) or permanent treatment discontinuation (7.0%).^36^

### Specific DILI management

As outlined in the general management of DILI section, a key step in diagnosing ChILI is the exclusion of alternative causes of liver test abnormalities, particularly progression of the underlying cancer, which has been widely described as the main reason for elevated aminotransferases among patients undergoing immunotherapy.^9^^,^^10^

International guidelines agree on the management of patients with grade 1 and 2 ChILI, with recommendations being to maintain ICIs for grade 1 hepatitis and temporarily discontinue ICIs until ALT normalisation or improvement for grade 1 hepatitis.^17^^,^^18^ According to these guidelines, all patients with grade 3 or 4 ChILI should permanently discontinue ICIs, and corticosteroids at a dose of 1-2 mg/kg/day should be initiated promptly.^17^^,^^18^ However, real-world data have shown that corticosteroids are not always needed. In two retrospective cohorts of patients with severe ChILI, 33%-50% of patients improved spontaneously after temporary interruption of ICIs.^16^^,^^54^ Lately, a prospective study that included 44 patients with either grade 3 or 4 ChILI showed that 67% of patients improve without corticosteroids according to a 2-step algorithm, based on temporary ICI discontinuation (step 1) and assessment of the degree of necroinflammation at liver biopsy (step 2).^10^

When corticosteroids are needed, doses of 1-1.5 mg/kg/day appear sufficient. In a retrospective cohort study of 215 cases of grade ≥3 ChILI, no differences in terms of time to ALT normalisation were observed when patients receiving methylprednisolone at ≥1.5 mg/kg were compared to those on lower doses.^55^ It is also important to consider that the use of corticosteroids in patients on immunotherapy may have a negative oncological impact and thus be detrimental in terms of survival.^56^ However, this statement is based on retrospective data, so larger and prospective studies are needed.

Another important consideration is that these patients are not eligible for LT if ALF develops and, for this reason, early identification of poor prognosis is crucial. Hence, some guidelines have suggested basing the management of severe ChILI on the total bilirubin and international normalised ratio (INR) values.^44^ For patients with severe ChILI but bilirubin <2.5 mg/dl and normal INR, temporary discontinuation of ICIs would be the first step, regardless of aminotransferase levels. In case of lack of improvement after 48-72 h, corticosteroids are recommended at a dose of 0.5-1 mg/kg/day, after ruling out alternative causes of hepatitis. In those patients presenting with increased levels of bilirubin (≥2.5 mg/dl), prompt therapy with corticosteroids at a dose of 1-2 mg/kg/day is highly recommended. In addition, in patients with an INR >1.5, a second immunosuppressive drug (tacrolimus or MMF), or even plasma exchange, should be considered.^57^^,^^58^

### Rechallenge after DILI

Overall, recurrence of irAEs is not common after rechallenge with ICIs. In a retrospective cohort including more than 6,000 patients who developed irAEs, the rate of relapse of any-degree irAE was 29%.^59^ Regarding ChILI, all guidelines agree on maintaining ICIs in grade 1 and grade 2 cases after improvement.^17^^,^^18^ In severe cases (CTCAE grade 3 and 4), international oncology guidelines recommend against rechallenge and endorse permanent discontinuation of immunotherapy.^17^^,^^18^ However, some retrospective real-world cohorts have shown that recurrence of ChILI is not universal. A multicentre prospective study including 23 patients with prior severe ChILI, according to the CTCAE criteria, reported a ChILI relapse rate of 34.8% following retreatment after aminotransferase levels had improved.^30^ Although the number of patients was relatively low, it is important to highlight that none of the patients with previous grade 4 ChILI recurred. More recently, in a cohort with 12 severe ChILI cases, no recurrences were reported after a median follow-up of 9 months.^60^

In this regard, it should be stressed that, as also reported for other severe irAEs, the more severe the ChILI, the better the oncological outcome.^61^^,^^62^ Therefore, for grade 3 and grade 4 ChILI, the possibility of rechallenge with ICIs should be assessed individually, considering not only the potential risk of rechallenge but also the therapeutic alternatives and the clinical benefit of maintaining ICI-based therapy.^44^

## Antibody-drug conjugates

### Mechanism of action and approved indications

**Fig. 2:**
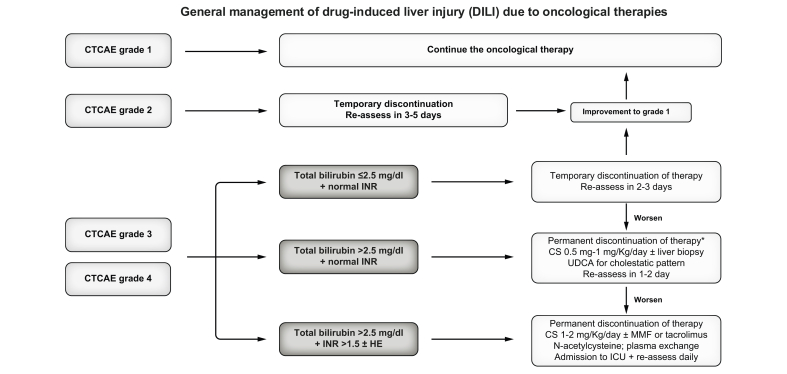
General management of DILI due to oncological therapies. For patients with grade 1 hepatitis (CTCAE) no changes are needed, and the causative drug can be maintained. For grade 2 hepatitis, temporary discontinuation is recommended until ALT normalises or improves to grade 1. For grade 3 or grade 4 hepatitis, management is based on total bilirubin and INR values. For patients with total bilirubin ≤2.5 mg/dl and INR <1.5, temporary discontinuation and work-up for alternative diagnoses is recommended, with follow-up every 2-3 days until ALT improvement. For those with bilirubin >2.5 mg/dl, prompt therapy with corticosteroids and daily follow-up are highly recommended. Second-line immunosuppressive agents such as MMF or tacrolimus are recommended in cases of insufficient response to corticosteroids. Moreover, UDCA may be useful in patients with a cholestatic pattern of injury. In those presenting with high bilirubin values but normal INR, the possibility of rechallenge could be individualised (∗). In those with coagulopathy or even acute liver failure, the use of support therapy with N-acetylcysteine or even plasma exchange could play a role since liver transplantation is contraindicated. ALT, alanine aminotransferase; CS, corticosteroids; HE, hepatic encephalopathy; ICU, intensive care unit; INR, international normalised ratio; MMF, mycophenolate mofetil; UDCA, ursodeoxycholic acid.

Since the approval of the first-generation ADC trastuzumab emtansine (T-DM1) for HER2+ breast cancer in 2013, subsequent generations of ADCs have significantly broadened the therapeutic landscape for advanced or metastatic breast, ovarian, urothelial, gastric and colorectal cancers (Table S3).^53^ Numerous clinical trials are currently investigating their application in earlier treatment lines and in combination with other anticancer modalities, such as ICIs.^54^ Among recent advances, mirvetuximab soravtansine has shown clinical benefit in platinum-resistant ovarian cancer^55^ and is emerging as a promising combination partner for bevacizumab.^56^^,^^57^ Similarly, enfortumab vedotin and tisotumab vedotin are demonstrating notable activity in urothelial and other solid tumours. Mechanistically, ADCs function through selective binding to target antigens on tumour cells, followed by internalisation of the ADC–antigen complex via endocytosis. This complex is trafficked through early and late endosomes before fusion with lysosomes, where proteolytic degradation of the ADC occurs, releasing the cytotoxic payload. The released drug subsequently induces apoptosis, typically via microtubule disruption or DNA intercalation (Fig. 2). HER2 is overexpressed in a considerable subset of breast, ovarian, and gastric cancers, establishing it as a key target for intervention. As a type I transmembrane glycoprotein, HER2 comprises an extracellular domain, a single-pass transmembrane region, and an intracellular tyrosine kinase domain, each essential for signal transduction. Monoclonal antibodies targeting the extracellular domain, such as trastuzumab, inhibit dimerisation within the HER family, thereby attenuating downstream signalling. Trastuzumab has demonstrated efficacy in both early and advanced HER2-positive breast cancer and is also approved for use in gastric cancer. Other ADCs of clinical interest include sacituzumab govitecan, which targets trophoblast cell-surface antigen 2. This conjugate links a humanised monoclonal antibody to govitecan, a topoisomerase I inhibitor, via a cleavable linker. Upon binding and internalisation, enzymatic cleavage releases the cytotoxic agent within the tumour cell. Likewise, mirvetuximab soravtansine-gynx, directed against folate receptor alpha, employs a similar strategy. It delivers DM4, a potent microtubule-disrupting agent, which is released intracellularly, causing cell cycle arrest and apoptosis. Enfortumab vedotin, which targets Nectin-4, and tisotumab vedotin, which targets tissue factor, follow a similar internalisation and release mechanism to deliver vedotin – a microtubule-binding cytotoxin that induces apoptosis. These ADCs represent an expanding class of precision therapies that harness targeted delivery with potent cytotoxicity to improve outcomes in a variety of malignancies.

### Incidence and DILI pattern

As summarised in a systematic review and meta-analysis, ADCs are associated with a high incidence of hepatotoxicity across all grades.^63^ Among patients receiving trastuzumab deruxtecan, the incidence of aminotransferase elevation of any grade, according to the FDA registration website, ranges from 10% to 38%, while only 1% or fewer patients experience aminotransferase levels of >5x the ULN. Real-world data suggest an incidence of liver toxicity of approximately 5%.^64^ In the registration trial of sacituzumab govitecan, elevations in serum aminotransferases were observed in 11% to 35% of treated patients; however, levels >5x the ULN occurred in only 1% to 2% of patients, with no reports of ALF.^65^ Liver toxicity is not among the most frequently reported adverse events associated with mirvetuximab soravtansine-gynx and, in some cases, is not documented at all.^65^ In an early phase I study, aminotransferase elevations of any grade were reported in 24% of patients for AST and 15% for ALT, while grade 3 elevations were observed in 2.2% of patients for both enzymes.^66^ A meta-analysis of studies on mirvetuximab reported grade ≥3 AST and ALT elevations in fewer than 5% of patients.^67^ In an early-phase trial of tisotumab vedotin,^68^ an elevation in ALT was observed in approximately 10% of patients. A subsequent review reported a higher incidence, with ALT increases occurring in up to 24% of cases, although none were >5x the ULN.^69^ Similarly, in a phase II study of enfortumab vedotin, ALT elevation was documented in 7% of participants, with no severe cases reported.^70^ While the mechanisms by which these agents induce liver injury have not been fully elucidated, they likely involve direct toxicity.

While trastuzumab alone, trastuzumab deruxtecan, sacituzumab govitecan, mirvetuximab soravtansine-gynx, tisotumab vedotin and enfortumab vedotin do not appear to be associated with severe liver injury or specific histological findings, nodular regenerative hyperplasia (NRH) has been linked to the use of T-DM1. NRH may develop after 1 year of therapy and is likely induced by the emtansine component.^71^^,^^72^

Interestingly, indirect signs of portal hypertension have been observed in patients treated with T-DM1, including splenomegaly, spontaneous porto-systemic shunts, and gastro-oesophageal varices, which may serve as useful markers for monitoring.^73^ Additionally, sinusoidal obstruction syndrome has been identified in liver biopsy specimens from patients receiving T-DM1, further confirming the vascular damage associated with this drug.^74^ In this line, gemtuzumab ozgamicin and inotuzumab ozogamicin, anti-CD33 and anti-CD22 antibodies, respectively, both used as treatments for acute myeloid leukaemia, have been associated with sinusoidal obstructive syndrome.^15^^,^^75^

### Specific DILI management

In cases of mild aminotransferase elevations (grade 1–2), close monitoring is generally sufficient, and dose adjustments are not typically required. However, for patients experiencing grade ≥3 elevations, dose reduction or treatment discontinuation is necessary. In most cases, these measures alone are sufficient to achieve normalisation of liver function tests.^76^ In instances of suspected immune-mediated hepatotoxicity, such as when ADCs are combined with ICIs, corticosteroids may be considered to mitigate liver inflammation. In cases of NRH, T-DM1 should be permanently discontinued.

### Rechallenge after DILI

The reintroduction of ADCs following a hepatic adverse event may be considered on a case-by-case basis. The safe reintroduction of trastuzumab following T-DM1-induced NRH has been documented in a case report.^77^

## Small molecule protein kinase inhibitor

### Mechanism of action and approved indications

Kinase inhibitors are a cornerstone of targeted cancer therapy, offering a more precise approach to treatment compared to traditional chemotherapies. These agents work by interfering with the activity of specific protein kinases – enzymes that regulate critical signalling pathways involved in cell growth, proliferation, survival, and differentiation. In cancer, many of these pathways become dysregulated due to genetic mutations, overexpression, or chromosomal rearrangements, leading to uncontrolled cell division and tumour progression.^78^ Tyrosine kinase inhibitors, one of the most widely used classes, target the phosphorylation activity of receptor and non-receptor tyrosine kinases, such as EGFR, HER2, BCR-ABL, ALK, ROS1, and VEGFR. By occupying the ATP-binding site of the kinase domain, these drugs block the activation of downstream signalling cascades like the MAPK and PI3K/AKT pathways, thereby inhibiting cellular proliferation and promoting apoptosis. In addition to tyrosine kinase inhibitors, other kinase inhibitors target serine/threonine kinases such as BRAF or cyclin-dependent kinases (*e.g.* CDKi). These agents disrupt cell cycle progression, particularly at the G1/S transition, leading to cell cycle arrest and eventual tumour regression. As of now, more than 80 kinase inhibitors have received FDA approval, 69 of them for cancer;^79^ the most important for solid malignancies are summarised in Table S4, while those with higher likelihood of inducing hepatotoxicity are listed in Table 1. PKIs represent the advancement of precision oncology based on the genetic alterations of individual patients.^77^

**Table 1:** FDA- and EMA-approved kinase inhibitors for solid tumours with high likelihood of DILI.

Drug target	Drug	Approved indications	DILI likelihood score#
BCR-Abl	Imatinib	GIST	B
CSF1, FLT3	Pexidartinib	Tenosynovial giant cell tumour	B
EGFR family	Erlotinib, Gefitinib	NSCLC, pancreatic cancer	B
	Lapatinib	Breast cancer	B
PDGFR	Gefitinib	NSCLC	B
	Imatinib	GIST	B
	Sorafenib	Renal cell carcinoma Hepatocellular carcinoma Thyroid cancer	B
	Regorafenib	Colorectal cancer and hepatocellular carcinoma	B
PDGFR, KIT	Sunitinib	Renal cell carcinoma	B

NSCLC, non-small cell lung cancer; GIST, gastrointestinal stromal tumours.

# Likelihood score indicates the likelihood of association with drug induced liver injury, based upon the known potential of the drug to cause such injury. According to Livertox https://www.ncbi.nlm.nih.gov/books/NBK548591/

**Table 2:** Rate, pattern of DILI and indications for the new oncological combinations associated with a high risk of developing DILI.

Combination	Family drugs	Main indication	Predominant DILI pattern	DILI rate	
				Any degree	Grade 3/4
Pembrolizumab OR avelumab + axitinib	Anti-PD1 or anti-PD-L1 + PKI (VEGFR)	Renal cell carcinoma	Hepatocellular	29%	20%
Nivolumab + cabozantinib	Anti-PD1 + PKI (VEGFR)	Renal cell carcinoma	Hepatocellular	25%	5%
Pembrolizumab + lenvatinib	Anti-PD1 + PKI (VEGFR)	Renal cell carcinoma	Hepatocellular	12%	4%
Atezolizumab OR pembrolizumab + bevacizumab	Anti-PD-L1 or anti-PD1+ anti-VEGF	Hepatocellular carcinoma	Hepatocellular	14%	4%
Pembrolizumab + enfortumab vedotin	Anti-PD1 + ADC (Nectin-4)	Urothelial carcinoma	Hepatocellular	3%	2%
Abemaciclib OR ribociclib OR palbociclib + exemestrane OR anastrozole OR letrozole OR tamoxifen OR fulvestrant	CDKi + endocrine therapy	HER2- breast cancer	Hepatocellular	20%∗	10%∗

ADC, antibody-drug conjugate; CDKi, cyclin-dependent kinase 4/6 inhibitor; DILI, drug-induced liver injury; PD-1, programmed cell death-1; PD-L1, programmed cell death-1 ligand; PKI, protein kinase inhibitor; VEGF(R), vascular endothelial growth factor (receptor).

∗ Rate of DILI is higher for abemaciclib and ribociclib than for palbociclib.

### Incidence and DILI pattern

A meta-analysis of 12 randomised-controlled trials demonstrated that patients receiving PKIs are at a significantly higher risk of developing high-grade hepatotoxicity compared to control groups.^80^ Interestingly, as demonstrated by a systematic review and meta-analysis encompassing nine trials and over 3,000 patients, newer-generation kinase inhibitors are more likely to induce elevations in aminotransferases compared to imatinib.^81^ However, the precise incidence of PKI-induced liver injury remains uncertain due to substantial variability depending on the specific agent involved.^82^ Moreover, emerging drugs have also been linked to hepatotoxicity, necessitating a degree of vigilance.^83^ This complexity is further compounded in cases where PKIs are administered in conjunction with ICIs. Hepatotoxicity associated with PKIs can manifest across a broad clinical spectrum, ranging from mild, asymptomatic elevations in liver enzymes to, in rare instances, ALF. The underlying pathogenic mechanisms include direct hepatocellular toxicity, mitochondrial dysfunction, and immune-mediated injury. It is postulated that PKI-induced liver damage primarily arises from the production of reactive metabolites and disturbances in endogenous metabolic pathways.^84^ The histopathological manifestations of PKI-related hepatotoxicity are highly heterogeneous. For instance, vemurafenib has been implicated in cases of granulomatous hepatitis,^85^ while immune-mediated hepatic injury has been reported in certain patients undergoing CDKi therapy.^21^ Sinusoidal congestion, necrosis of hepatocytes, and inflammation have been described in cases of imatinib toxicity,^86^ while inflammation predominantly involving portal tracts and bile duct epithelial injury were observed on biopsies from two patients treated with pazopanib.^87^

### Specific DILI management

Depending on the drug, the underlying mechanism of toxicity and the pattern of liver injury, different management strategies can be proposed. Besides dose reduction or discontinuation, corticosteroids have been successfully used in cases of immune-mediated DILI, such as toxicity from CDKi^21^ or sotorasib.^88^ However, to date, only retrospective and scarce data are available on the use of corticosteroids for CDKi-induced liver injury and, therefore, providing recommendations is challenging. Nevertheless, patients presenting with bilirubin of ≥2x ULN may benefit from corticosteroid therapy. In addition, N-acetylcysteine has also been used for CDKi-related hepatotoxicity.^89^ Although its efficacy has been demonstrated in non-acetaminophen ALF^90^ its role in managing non-severe cases remains to be established. It has been suggested that fenofibrate, a PPAR (peroxisome proliferator-activated receptor-α) agonist could significantly attenuate liver injury from sunitinib.^91^

### Rechallenge after DILI

Treatment reintroduction may be considered at lower doses and with weekly monitoring of liver tests. Rechallenge appears to be a feasible approach even with drugs of the same pharmacological class. This has been demonstrated in cases involving CDKi – for example, when palbociclib was administered after grade ≥3 liver toxicity with ribociclib^92^^,^^93^ – and EGFR kinase inhibitors, when genfitinib was introduced after erlotinib hepatotoxicity.^94^^,^^95^ Furthermore, the successful reintroduction of the same molecule following an episode of liver toxicity has also been documented. For instance, reintroduction of sotorasib, a KRAS G12C inhibitor, did not lead to recurrence of hepatotoxicity, suggesting that, with appropriate monitoring and management, continuation of treatment may be feasible even after an initial adverse hepatic event.^88^ Similarly, crizotinib, an ALK kinase inhibitor, was reintroduced at half the original dose following ALF induced by the same agent.^96^

## Combination regimens that carry a risk of DILI

The evolving use of oncological drug combinations is likely to increase the complexity of managing liver toxicity (Table 2). It is therefore essential for hepatologists to become familiar with these emerging regimens to ensure early recognition and appropriate management of hepatic adverse effects.

### Immunotherapy plus PKIs

Multiple combinations of anti-PD1 or anti-PD-L1 agents with a PKI have been tested in phase I and II trials for metastatic solid-organ tumours. To date, such combinations have been approved for advanced renal cancer, endometrial carcinoma and HCC.^97^

### Pembrolizumab or avelumab plus axitinib

Pembrolizumab plus axitinib demonstrated an improvement in terms of overall and progression-free survival compared to sunitinib in treatment-naïve patients with advanced renal cell carcinoma.^98^ However, 20.4% (87/426) of patients presented with grade-3/4 hepatitis and 4.2% with any-degree ALT increase plus a bilirubin level ≥2x the ULN (moderate severity based on IEWG). The majority of these patients recovered after treatment discontinuation, though corticosteroids were also used in 54% and 62% of grade 3/4 hepatitis or moderate (IEWG) severity hepatitis cases, respectively.^99^ Interestingly, for grade 2 and 3 hepatitis, no differences in time to ALT normalisation were observed among those managed with or without corticosteroids. After recovery, 100/120 patients were rechallenged, and the risk of grade 3/4 hepatitis recurrence varied according to the drug/combination used at retreatment: 29/37 (78.4%) axitinib, 1/4 (25.0%) pembrolizumab, 25/59 (42.4%) pembrolizumab-axitinib. No liver-related deaths were reported.^99^

### Nivolumab plus cabozantinib

The combination of nivolumab plus cabozantinib demonstrated superior progression-free and overall survival compared to sunitinib in patients with advanced renal cell carcinoma.^100^ Any-degree ALT increase was reported in 25% of patients, including 5% with grade 3 hepatitis and 1 (0.3%) case of ALF.^101^ The majority of patients who developed grade 3 hepatitis while receiving nivolumab plus cabozantinib were managed by interruption of the drugs plus corticosteroids.^100^^,^^102^

### Pembrolizumab plus lenvatinib

The phase III CLEAR study revealed longer progression-free and overall survival for pembrolizumab plus lenvatinib compared to sunitinib for patients with advanced renal cell carcinoma.^103^ In the registry study, the rates of any-degree ALT increase and grade 3 hepatitis were 12% and 4.3%, respectively – very similar percentages to those observed when lenvatinib was combined with everolimus.^103^ One case of ALF was reported in the pembrolizumab-lenvatinib cohort.

Regarding management of severe DILI due to an anti-PD1 or anti-PD-L1 agent plus a kinase inhibitor, firstly, the same diagnostic work-up as for patients with ChILI is recommended (Fig. 1), with prompt interruption of both drugs and follow-up every 48-72 h. Differences in the half-lives of PKIs *vs.* anti-PD1 or anti-PD-L1 agents may help in identifying the potential cause of hepatotoxicity. Cases that improve after 3-5 days of treatment discontinuation may implicate the PKI as the principal cause of toxicity. In cases of moderate IEWG severity, grade 4 hepatitis (CTCAE), or a lack of improvement despite temporary discontinuation of both drugs, corticosteroids should be administered. Close monitoring is recommended until ALT normalisation or a decrease to grade 1.

Cases of moderate IEWG severity should not be rechallenged with the combination. Otherwise, the decision regarding retreatment and which drugs to use should be individualised based on the severity of the initial DILI episode, the suspected causative drug and the oncological benefit of the combination.

## Immunotherapy plus anti-VEGF

### Atezolizumab or pembrolizumab plus bevacizumab

The phase III IMbrave150 trial demonstrated that atezolizumab plus bevacizumab, a monoclonal antibody that targets tumour vascular endothelial growth factor (VEGF), improved overall and progression-free survival compared to sorafenib, leading to the approval of this combination as the first-line standard of care for unresectable HCC.^104^ The overall rates of ALT increase and grade 3/4 hepatitis among patients on atezolizumab–bevacizumab were 14% and 3.6%, respectively.^104^

Bevacizumab seems not to be associated with liver injury, rather, it has been proposed that bevacizumab may play a protective role against the liver injury caused by other chemotherapeutic agents, such as oxaliplatin.^105^ Thus, management recommendations for patients receiving atezolizumab–bevacizumab may be extrapolated from those for ChILI, with consideration given to maintaining bevacizumab if no concomitant adverse event related to the anti-VEGF agent is present.

### Immunotherapy plus an antibody-drug conjugate

Combinations of ADCs plus ICIs have been evaluated due to their non-overlapping mechanisms and potential to overcome the resistance observed with single-agent therapy.^106^ Urothelial carcinoma was the first advanced tumour in which these combinations were successfully tested and shown to have an acceptable safety profile.

### Pembrolizumab plus enfortumab vedotin

The combination of pembrolizumab plus enfortumab vedotin has shown superiority to platinum-based chemotherapy in patients with previously untreated locally advanced or metastatic urothelial carcinoma.^107^ Any-degree and grade 3 hepatitis were reported in 3.2% and 1.8% of patients in the pembrolizumab-enfortumab vedotin arm, compared to 0.5% and 0% in the chemotherapy arm. No cases of hepatitis led to treatment discontinuation. Though no comparative trials have been performed so far, it seems that the DILI rate may be higher among patients receiving the pembrolizumab-enfortumab vedotin combination than among those on enfortumab vedotin alone. In view of the lack of evidence, cases of hepatotoxicity should be managed as recommended for patients with ChILI.

## PKIs plus other drugs

### CDK4/6 inhibitors plus endocrine therapy

CDKi have become the first-line standard treatment for most patients with HER2-metastatic breast cancer and, more recently, ribociclib and abemaciclib have received approval as adjuvant therapies for women with high-risk early breast cancer.^108^^,^^109^

In both contexts, CDKi are combined with endocrine therapy. Ribociclib and abemaciclib are combined with an aromatase inhibitor (exemestrane, anastrozole or letrozole) or tamoxifen (abemacilib) in early disease, while the standard combinations include aromatase inhibitors or fulvestrant in advanced breast cancer.

All aromatase inhibitors have been associated with ALT increases. For instance, in a phase III study that included 371 postmenopausal women with metastatic breast cancer, grade 3 hepatitis was observed in 7.7% of patients treated with exemestane and 4.2% of those on tamoxifen.^110^

It is important to mention that tamoxifen has also been linked with fatty liver disease, steatohepatitis, cirrhosis and HCC,^111^ whereas other endocrine therapy drugs have not.

The incidence of DILI is clearly higher in patients receiving endocrine therapy combined with a CDKi, as shown in the pivotal phase III study of ribociclib, where grade 3 hepatitis was reported in 10.4% of patients receiving both ribociclib and letrozole, in comparison to 1.2% of those on letrozole alone.^112^ In case of grade 2 hepatitis or higher, guidelines recommend the discontinuation of the CDKi, without suggesting what should be done with regards to the endocrine therapy. As stated in the DILI general management section, in cases of severe DILI, temporary discontinuation of all potential hepatotoxic drugs is recommended. However, in daily clinical practice, toxicity is normally attributed to the CDKi, so the endocrine therapy may be maintained during CDKi discontinuation. To date, there is no evidence on the potential impact of discontinuing both drugs compared to the CDKi alone on the time to ALT normalisation, though it is possible that the temporary cessation of both drugs would lead to more rapid normalisation.

## Conclusions

In summary, improvements in cancer treatment have led to longer patient survival but also higher rates of toxicity. The mechanisms underlying hepatotoxicity caused by these new compounds remain poorly understood, and there is a great need for data on histology, management, and the risk of rechallenge to ensure patient safety. The approval of new adjuvant therapies to reduce the risk of relapse of resectable tumours will increase the need for a multidisciplinary approach and individualised decision making in the event of liver injury, as the balance between safety and oncological benefit is even harder to determine.

## Abbreviations

ADC, antibody-drug conjugate; ALF, acute liver failure; ALT, alanine aminotransferase; AST, aspartate aminotransferase; CDKi, cyclin-dependent kinases 4/6 inhibitor; ChILI, checkpoint inhibitor-induced liver toxicity; CTLA-4, cytotoxic T-lymphocyte antigen 4; DILI, drug-induced liver injury; GIST, gastrointestinal stromal tumour; HCC, hepatocellular carcinoma; HER2, human epidermal growth factor receptor 2; ICI, immune checkpoint inhibitor; IEWG, International DILI Expert Working Group; irAEs, immune-related adverse events; LT, liver transplantation; MMF, mycophenolate mofetil; NRH, nodular regenerative hyperplasia; NSCLC, non-small cell lung cancer; PD1, programmed cell death-1; PD-L1, programmed cell death-1 ligand; PKI, protein kinase inhibitor; T-DM1, trastuzumab emtansine; ULN, upper limit of normal; VEGF, tumour vascular endothelial growth factor.

## Financial support

The authors received no specific funding for this work.

## Authors’ contributions

Mar Riveiro-Barciela: conceptualization; writing – original draft; investigation. Eleonora De Martin: conceptualization; writing – original draft; investigation.

## Conflict of interest

The authors declare that they have no affiliations with or involvement in any organization or entity with any financial interest in the subject matter or materials discussed in this manuscript.

Please refer to the accompanying ICMJE disclosure forms for further details.
